# Identification of Local Conformational Similarity in Structurally Variable Regions of Homologous Proteins Using Protein Blocks

**DOI:** 10.1371/journal.pone.0017826

**Published:** 2011-03-18

**Authors:** Garima Agarwal, Swapnil Mahajan, Narayanaswamy Srinivasan, Alexandre G. de Brevern

**Affiliations:** 1 Molecular Biophysics Unit, Indian Institute of Science, Bangalore, India; 2 National Centre for Biological Sciences, Tata Institute of Fundamental Research, UAS-GKVK Campus, Bangalore, India; 3 Dynamique des Structures et Interactions des Macromolécules Biologiques (DSIMB), INSERM, U665, Paris, France; 4 Université Paris Diderot - Paris 7, UMR-S665, Paris, France; 5 Institut National de la Transfusion Sanguine (INTS), Paris, France; University of Stellenbosch, South Africa

## Abstract

Structure comparison tools can be used to align related protein structures to identify structurally conserved and variable regions and to infer functional and evolutionary relationships. While the conserved regions often superimpose well, the variable regions appear non superimposable. Differences in homologous protein structures are thought to be due to evolutionary plasticity to accommodate diverged sequences during evolution. One of the kinds of differences between 3-D structures of homologous proteins is rigid body displacement. A glaring example is not well superimposed equivalent regions of homologous proteins corresponding to α-helical conformation with different spatial orientations. In a rigid body superimposition, these regions would appear variable although they may contain local similarity. Also, due to high spatial deviation in the variable region, one-to-one correspondence at the residue level cannot be determined accurately. Another kind of difference is conformational variability and the most common example is topologically equivalent loops of two homologues but with different conformations. In the current study, we present a refined view of the “structurally variable” regions which may contain local similarity obscured in global alignment of homologous protein structures. As structural alphabet is able to describe local structures of proteins precisely through Protein Blocks approach, conformational similarity has been identified in a substantial number of ‘variable’ regions in a large data set of protein structural alignments; optimal residue-residue equivalences could be achieved on the basis of Protein Blocks which led to improved local alignments. Also, through an example, we have demonstrated how the additional information on local backbone structures through protein blocks can aid in comparative modeling of a loop region. In addition, understanding on sequence-structure relationships can be enhanced through our approach. This has been illustrated through examples where the equivalent regions in homologous protein structures share sequence similarity to varied extent but do not preserve local structure.

## Introduction

Comparison of protein structures is an indispensable step in understanding structure-function relationships. In most cases, first reasonable impressions on function of a protein can be generated if the protein shares high structural similarity to a protein of known function [1]. It also gives hint on evolutionary relationships [2]–[5]. During evolution, fold of homologous proteins are conserved even without detectable sequence similarity [6], [7]. High structural similarity associated to the very low sequence similarity is indicative of either a common origin [4], [6] or an independent origin with convergence to a common fold [8]. During evolutionary process, different regions of proteins are constrained differently; the regions critical for functional and structural integrity are well preserved, while the rest of the structure can diversify to accommodate insertions, deletions and substitutions [9], [10].

The 3-D superimposition of protein structures obtained by using structure comparison tools is very useful in quantifying structural dissimilarity and in analyzing structural divergence. Structural comparison influences classification of proteins into protein families, superfamilies etc [11],[12], *i*.*e*., they allow a complete representation of protein fold space. Hence, for a newly determined protein structure, mining the structural databases enables the identification of protein structures/sub-structures similar to the given structure [13]–[18]. Proteins are not rigid macromolecules and they exhibit certain degree of flexibility to allow structural variations critical for functional mechanisms [19]. Thus comparison of structures corresponding to the active and inactive states of a protein can further our understanding on the conformational plasticity of protein structures and the insights gained can improve the drug design process [20]–[22].

Alignment of proteins on the basis of their 3-D structures is more complex than sequence-based alignment as the 3-D structural information is more complex [23]. From a computational point of view, identifying the best match having least spatial distance between the maximum numbers of equivalent regions is highly expensive. Heuristics is usually added to make the problem tractable. The difficulty in aligning structures is compounded when the structures share similar secondary structures with different connectivity. In such cases, matching of equivalent regions is not sequential. Due to its utility and the difficulties mentioned before, innumerable methods to compare and align protein structures have been developed, *e*.*g*., DALI [14], SSAP [24], MAMMOTH [25], CE [26], COMPARER [27], FATCAT [28] and Matt [29]. These methods seek to find maximum correspondences between the structural elements (*i*.*e*., atoms, residues or secondary structures), and compute a similarity measure. They differ at the level of (i) representation of protein structure (points, vectors, internal distances or graphs), (ii) measure of similarity and (iii) the algorithm used for comparison, (for reviews see [30]–[32]). The comparison algorithms are varied such as dynamic programming, stochastic algorithms like Monte Carlo methods and graph theory based methods.

The algorithms can be grouped into rigid body methods, that view protein structures as rigid bodies, *e*.*g*., STAMP [33], DALI [14], CE [26] and MAMMOTH [25] and flexible methods, that connect series of aligned fragments or substructures, *e*.*g*., FATCAT [28], FlexProt [34] and Matt [29]. The structural alignments provided by flexible methods is believed to be better as they are biologically more meaningful [35].

In this work, we attempt to add a component of flexible alignment in local variable regions which are initially recognized by rigid body superposition. Here, we focus on the “structurally variable” (high spatial deviation) regions in the alignments of three-dimensional structures of homologous protein domains in PALI database [36]. PALI database comprises of protein families from SCOP [11]. It contains structural alignments generated using DALI [14], a well established structure comparison method and subsequently superimposed using rigid body alignment method. After such a rigid body superposition, the backbone regions with highly similar structures are evident by good overlap of Cα atoms. The structural differences in homologous proteins could be due to structural re-ordering to accommodate mutations. These differences vary from subtle variations in backbone structure to large orientation differences to accommodate substitutions especially at the core [6], [37]–[39]. Insertions are accommodated as an extension to the existing secondary structures or addition of new regular/irregular structures [37], [38], [40]. These insertions may either act as embellishments or promote functional diversity by presenting altered/new binding site for ligand or macromolecule [38], [40].

The current study pertains to those backbone regions of homologous proteins that are not well superimposed in the rigid body superposition. These structural differences between homologues can be categorized into rigid body displacements and conformational variations. However due to rigid body displacements, an optimal superposition may not be obtained using a rigid body superposition method. Through Protein Blocks [41], a simplified representation of protein structures, we classify the variable regions into conformationally dissimilar regions and regions that share local structural similarity obscured in a global fit. In the next step we refine the alignment between homologues in PALI database, obtained through a recognized rigid body method, using match of protein blocks in the local structurally variable regions. Additionally, based on the similarity measure used, the assignment of residue-residue equivalences for a structural superposition may differ [23]. The discrepancies are higher when the Cα-Cα deviation is high. An optimal local alignment would help in the assignment of residue-residue equivalences more precisely.

For this work, Protein Blocks (PBs) [41]–[44] is the major tool used. They represent a higher level abstraction of protein backbone conformation. This is a set of 16 prototype conformers, denoted from *a* to *p*, which approximate the local protein structure with an average root mean square deviation of 0.42 Å. Protein Blocks have been used in comparison of protein structures [41], [45] and database mining [46]. PBs have been found to be useful in prediction of short loops [47]. Protein blocks approach has also been used to build trans-membrane protein structures [42], to design peptides [48], to define reduced alphabets for designing mutants [49], to analyze protein contacts [50], to find structural motifs across protein families [18] and to identify Mg^2+^ binding sites in proteins [51].

## Results and Discussion

Superimposed proteins from PALI database have regions of correspondence that exhibit high structural deviation, namely “Structurally Variable Regions” (SVRs). These regions may appear “structurally variable” (not well superimposed) in a global context but may exhibit local conformational similarity. For example an α-helical region in a protein might correspond to an α-helical region in the homologue; however if the helical regions in the two proteins are in slightly different orientations they may not appear superimposed if the two structures are superimposed as a whole. Using PB Substitution Matrix (SM) coupled with CLUSTALW [52] alignment approach, SVRs were re-aligned to seek an improvement in the local alignment for these regions (see *Materials and Methods* section). We investigated the differences in alignments obtained after employing protein blocks approach (aSVRs – “a” stands for “after”) and alignments before employing the approach (bSVRs – “b” stands for “before”), to evaluate our protocol in revealing similarities not identified using a global rigid-body superposition method. For this purpose, we compared the two alignments, referred to as bSVRs and aSVRs in the rest of this paper, based on PB scores and values of root mean square deviation (rmsd) or a similar measure, Structural Distance Metric (SDM). An improvement in the values for these two parameters for aSVR would reflect an improvement in the alignment obtained using PBs. A total of 347,062 Structurally Variable Regions (SVRs) and 542,610 Structurally Conserved Regions (SCRs) were identified in the PALI database (Refer *Materials and Methods*).

### Distribution of scores

**Figure 1:**
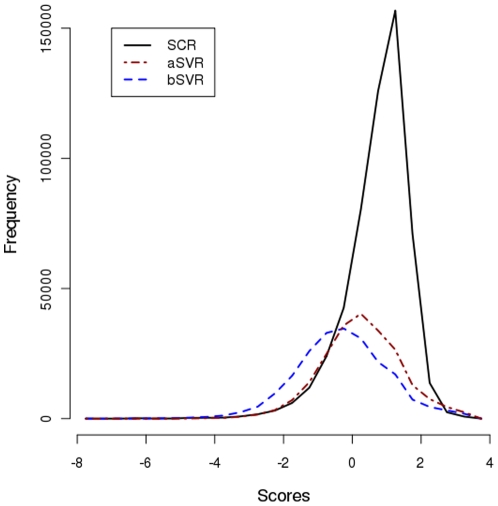
Distribution of scores for aligned PBs in structurally conserved regions (SCRs, solid line), structurally variable regions before re-alignment (bSVRs, broken lines,blue) and SVRs which have been re-aligned using PB approach (aSVRs, red).

Re-alignment of PB sequences of SVRs change the alignment scores. Figure 1 shows the distribution of normalized score for aligned pairs (SAP score) obtained for SCRs, bSVRs and aSVRs. A normal distribution of scores was observed. Using two-sided Kolmogorov-Smirnov statistic, the p value for each of the three distributions is less than 2.2e-16. As expected, the values for SAP are higher in SCRs as compared to bSVRs indicating higher structural similarity in SCRs compared to bSVRs. However, compared to bSVRs, a significant shift of SAP values towards higher scores was observed for aSVRs (p value <2.2e-16; Paired student *t* test, see Figure 1). An analysis of the difference in SAP values for aSVRs and bSVRs indicates an improvement for 56% of SVRs and a decrease for 13% of SVRs. The scores remain unchanged for the remaining 31% of the alignments. The trend for the distribution of scores for complete alignment (SCA) was similar to SAP scores; 59% aSVRs scored higher and 14% scored lower than bSVRs (see Text S1 and Figure S1). SCA and SAP scores have reasonable correlation in the two scores for both bSVRs and aSVRs. A shift could be observed towards higher scores; 55% of aSVRs scored above -1 for SCA and SAP measurements as opposed to 41% of bSVRs, *i*.*e*., an increase in number by 14% (see Text S2 and Figure S2). Thus, an increase in the scores after realignment indicates that the regions concerned are more similar in terms of conformation than previously represented in the PALI database. Both measurements show improvement indicating better alignment of SVRs.

Analysis of the distribution of SCA and SAP allowed us to define a cutoff score of −0.42 to distinguish SVRs as conformationally similar and dissimilar (see Figure 1). The cutoff was chosen such that 90% of the scores corresponding to the structurally conserved regions score above this threshold. Based on this cutoff, 53% of the bSVRs and 74% of aSVRs were classified as conformationally similar, *i*.*e*., an increase by 21% (45,343 SVR segments). Thus, through our approach we have been able to identify local structural similarity in a substantial number of SVRs which was not known from classical approach.

### PB substitutions in bSVRs and aSVRs

**Figure 2:**
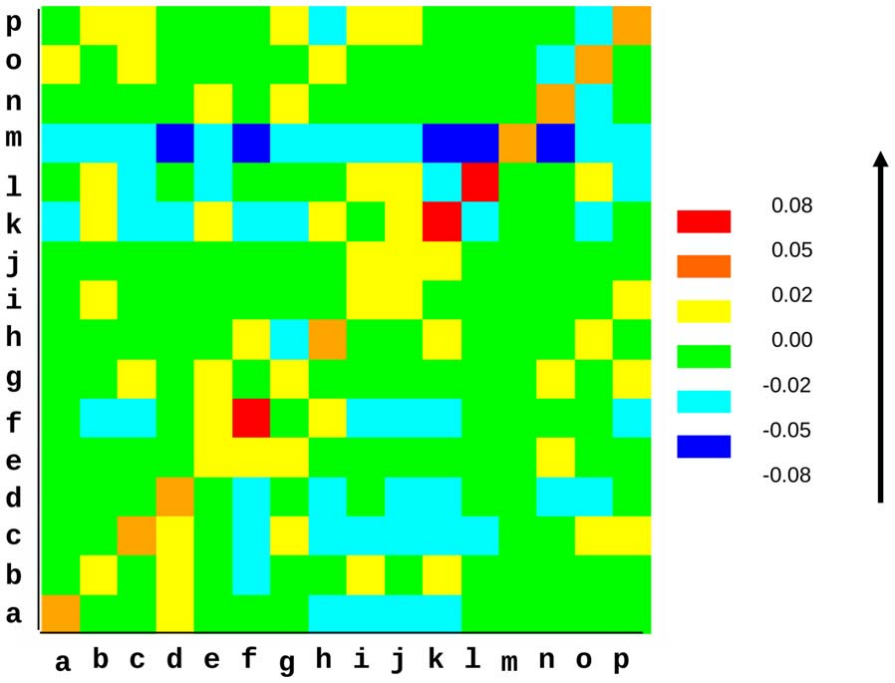
Difference in distribution of nature of aligned PBs observed after re-alignment of SVRs compared to the original alignment. Various colors indicate the extent of differences in the number of various PB-PB equivalences between bSVRs and aSVRs. Blue color indicates a decrease and red color indicates an increase in the number of corresponding PB equivalences in aSVRs compared to bSVRs. The other colors indicate intermediate values.The values in the top diagonal of the matrix have been normalized by the number of PBs as denoted in x axis. Similarly, the values in the bottom diagonal have been normalized by the number of PBs as denoted in the y axis.

An improvement of scores is observed after re-alignment. This increase is due to a higher number of PB-PB equivalences (*i*.*e*., number of PB aligned with another PB and not a gap), and/or a change in the nature of PBs aligned at various positions in the alignment. 76% of SVRs showed no change in the raw number of correspondences after re-alignment. On an average, for each segment, 0.5 more PB is aligned with a PB in aSVRs as compared to bSVRs (see Figure S3). Figure 2 shows the difference in the distribution of PB-PB substitutions between bSVRs and aSVRs. Alignment of identical PBs (*i*.*e*., diagonal elements of the plot) is increased for each PB. Among the non-identical substitutions, the highest increase has been observed for the alignments of PB *f* (C cap β strand), PB *k* and *l* (loop to N cap α helix). A lower increase was observed in the alignments of PB *a* and *c* (N cap β strand), PB *d* (β strand), PB *m* (α helix), PBs *n* to *p* (C cap α helix) and PB *h* (loop). The alignments of PB *m* with each PB types except itself shows a drop, the highest decrease being in the alignments with PBs *d*, *f*, *k*, *l* and *n*. A lower decrease was observed for the alignment of PB *a* and *c* with PBs corresponding to loops, PB *d* with PBs corresponding to loops and capping regions of α helix, PB *f* (C cap β strand) with PBs corresponding to N cap β strand and loops, PB *k* with PBs corresponding to N and C caps of β strand and PBs *l* with PBs *c*, *e*, *k* and *p*. In general, this decrease concerns unrelated or dissimilar PBs and the increase is mainly observed in highly similar or identical PBs. The increase in the number of equivalences for PBs corresponding to N cap and C cap regions of helices and strands as seen in Figure 2 suggests an improved alignment of these regions. Similar conclusions were drawn from the plots generated for data sets corresponding to various SCOP classes.

Hence, the major contributing factor for the increase in scores is the change in the type of equivalences rather than an increase in the number of correspondences. In fact only 30% of SVRs share more than 95% of the equivalences. In the rest 70% of SVRs (see Figure S4A) the percentage of equivalence is shared to varied extent. Nevertheless, a common PB pair found in the two alignments could in fact come from different regions in the sequences. 40% of SVRs have undergone changes in the alignments to form new equivalences although the PB-PB equivalences are preserved, while for 40% SVRs, the equivalences are retained in the alignment (see Figure S4B).

A comparison of the difference in percentage of gaps between bSVRs and aSVRs (Figure S5A) shows that 76.6% of the alignments have no change in the number of gaps. A decrease in the percentage number of gaps has been observed for 18.4% SVRs and an increase is seen in 5.0% of SVRs. Although, a decrease in the percentage of gaps is indicative of higher similarity in terms of lengths of the protein structures aligned, the introduction of gaps is sometimes favored as it reduces the number of equivalences of dissimilar PBs. Another interesting parameter compared was the number of gap openings in the alignments. An accommodation of insertions and deletion would require a re-adjustment in protein structures. We would expect fewer insertion and deletion events during protein evolution to preserve the three dimensional structure and thus intuitively less number of gaps interspersed in the alignments especially in the middle of helices and strands [37], [38], [53]. The difference in the number of gap openings in aSVRs as compared to bSVRs is not significant (mean value equals to −0.38) (see Figure S5B). Nonetheless, some examples were observed where gaps in the stretch of aligned PBs corresponding to α-helix and β-strand are eliminated in aSVRs (see the section below).

### Analysis of SVRs

Local structural similarity could be identified in terms of PB sequence similarity. We have also analyzed it by comparing SDM of bSVR and aSVR alignments. Profit software [54] was used to perform the superimposition. *Rmsds* obtained from these superimpositions were converted in SDMs (Structural Distance Metric) [55], [56] (see *Materials and methods* section).

With a global rigid body protein structure superposition, regions corresponding high deviations usually correspond to regions (i) that are spatially displaced although being structurally similar or (ii) with genuine difference in local conformation. Protein Blocks approach can distinguish these two scenarios. Indeed, a rigid body displacement of a local region after superposition would result in a high PB score and low SDM for the aligned regions. However, when the regions are conformationally distinct, the PB score would be low and SDM would be high.

**Figure 3:**
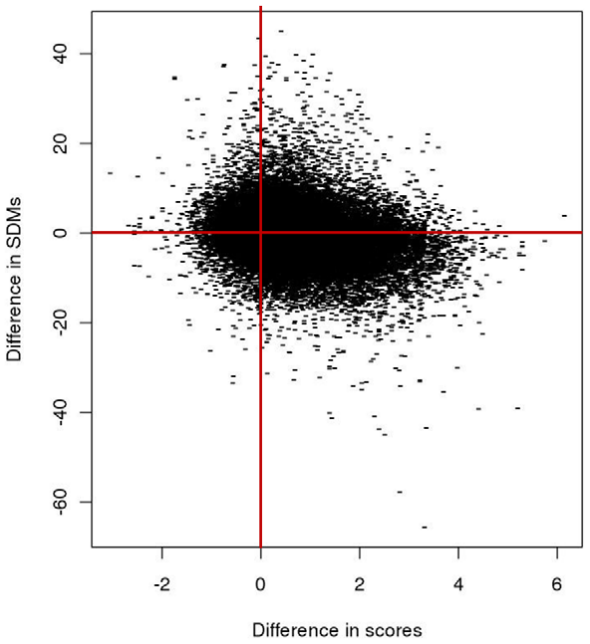
Difference in scores of aligned PBs (SAP score) and of SDM values between aSVRs and bSVRs. A negative difference in SDM values and a positive difference in scores indicate an improvement.

Figure 3 shows a plot indicating the variation of difference in SDM values with respect to the difference in PB SAP scores for segments before and after re-alignment, *i*.*e*., aSVRs and bSVRs.

**Figure 4:**
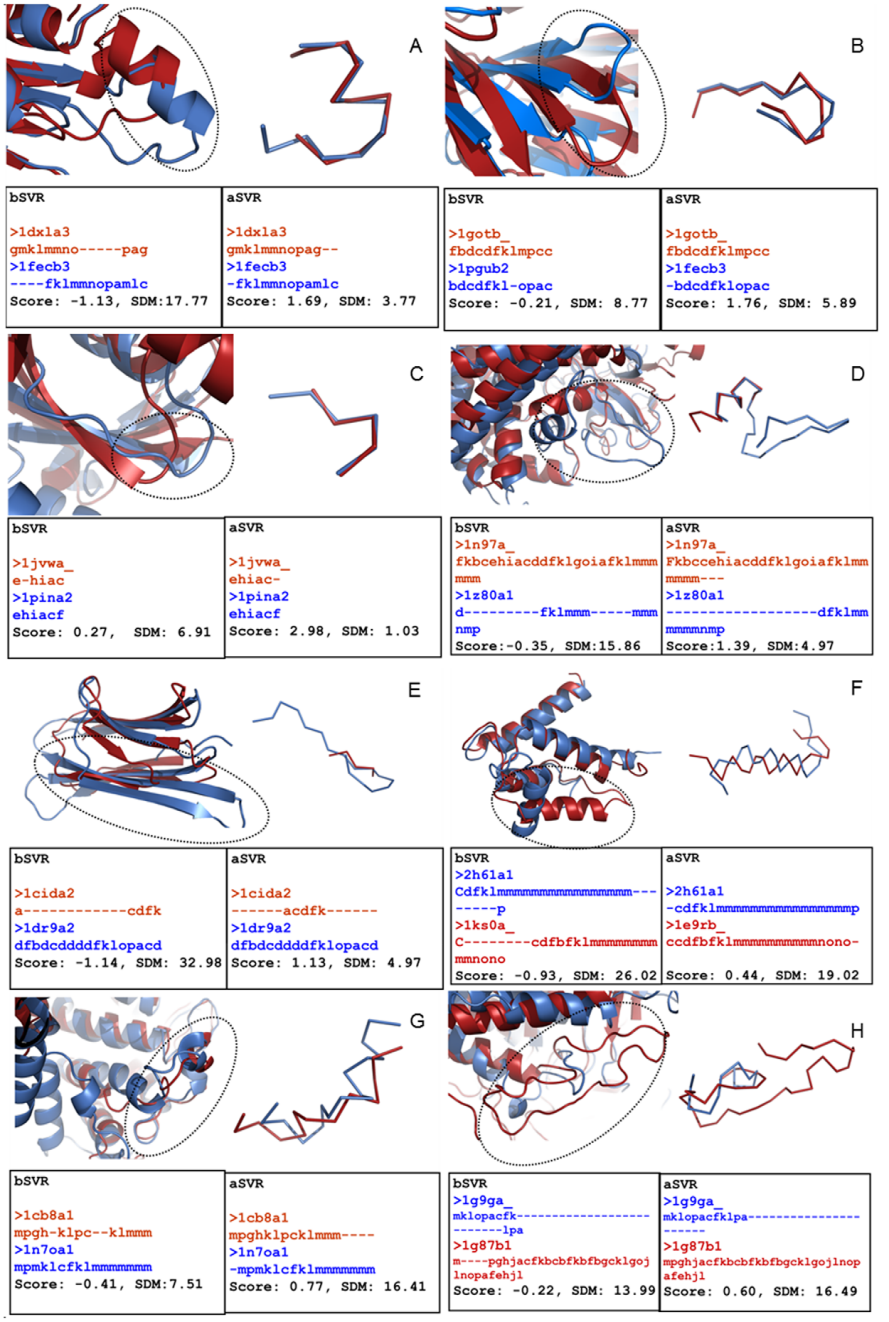
Illustrative examples of superposition of SVRs before and after realignment using PB approach. The global superposition of protein structures before re-alignment is shown as cartoons in blue and red. The regions which were re-aligned locally are encircled. The local superposition is shown in ribbon representation. PB equivalences, scores and SDM values for bSVRs and aSVRs are also shown. This Figure and other figures showing an overlay of protein structures have been generated using Pymol software [82]. A–F: Examples of improved PB scores and SDM values using PB approach. G and H: Improved PB scores but an increase in SDM. Refer text for further details.

The results on the assessment of approach in terms of SDM and PB scores have been tabulated (see Table 1). On an average 36.2% of SVRs showed an improvement in SDM values. For 32.7% no change has been observed while for 31.0% a decrease has been observed. In the last category of cases, often superimposition is not relevant as the mean PB scores for these SVRs is 0.02 after re-alignment (−0.49 before re- alignment). 28.9% of the SVRs in the dataset showed an improvement both in PB scores as well as SDM values. Improvements were due to re-alignment of segments which were displaced/oriented differently in previous alignments. Figure 4A shows an illustrative example highlighting improved alignment of an α-helix displaced in alignment obtained by superposition of gross structures. The figure on the left shows a superposition of SVR segments based on alignment obtained by using DALI. Superposition of the segments obtained after re-alignment is shown on the right. Below each superposition, are shown the alignments, PB scores and SDM values for alignments, bSVRs and aSVRs. With appropriate placement of gaps, PBs *k*, *l*, *m*, *n*, *o*, *p* and *a* are aligned in aSVR thus identifying local structural similarity previously unknown. Similarly, a β-strand oriented differently in the homologue could be aligned with a lower SDM using PB approach as shown in the Figure 4B. Figure 4C shows the superposition of regions corresponding to loops. Although the loop conformations are identical, as indicated by the identical PBs in the two structures, the SDM is high due to difference in the orientation (superposition on the left). An optimal superimposition and residue-residue equivalences could be obtained using PB approach (superposition on the right). As exemplified above, the local structural similarity was unidentified previously due to rigid body shifts. In other cases, improvements were observed in alignment of segments with differing lengths but with local structural similarity. The region of similarity was found to lie at either ends in the alignments or in the middle of alignment flanked by gaps. Figure 4D shows an example of a region similar at one end. Moreover, the continuity in helix in the new alignment is evident bringing the PBs *f*, *k*, *l and* series of *m* in the two sequences in register. As mentioned in the previous section, insertions and deletions in the middle of a helix or a strand are tolerated to a lesser extent as compared to the rest of the structure. Through our approach, gaps in the middle of a helix or a strand have been reduced/eliminated. Figure 4E shows an example of a region of local similarity in the middle of alignment. The PBs *a*, *c* and *d* correspond to a small strand with a transition to coil-like region denoted by PBs *k* and *l* in the variable segment of CD4 glycoprotein (PDB code: 1cid, chain A; shown in red; [57]). This region is aligned with the C terminal end of the β-strand in the homologue (in blue). The example highlights an improvement in the capping region of β-strand transiting to coils. The improvement in scores could also be attributed to a decrease in the equivalences of PBs corresponding to PB *m* (*i*.*e*., helical state) with PBs associated to strands and capping regions (*i*.*e*., PBs *d*, *b*, *c* and *f*,) as illustrated in Figure 4F. An alignment of a helix and a strand is meaningless in structural context as these regions, though equivalent in homologous proteins, do not share structural similarity. Hence the alignment of PBs corresponding to helices and strands would be insignificant.

**Table 1:** Results on assessment of aSVRs in terms of PB scores and SDM*.

		ΔSDM			
		better	equal	Worse	Sum
**ΔSCA**	**better**	19457 (28.9)	12 (0.02)	14654 (21.8)	**34,123 (50.8)**
	**equal**	1158 (1.7)	21989 (32.7)	1024 (1.5)	**24,171 (36.0)**
	**worse**	3755 (5.6)	2 (0.003)	5183 (7.7)	**8,940 (13.2)**
	**Sum**	**24370 (36.2)**	**22003 (32.7)**	**20861 (31.0)**	**67,234 (100.0)**

*The numbers outside brackets correspond to the number of SVRs. These numbers expressed as percentage are shown in brackets.

32.7% of the alignments showed no difference in PB scores and SDM. The plausible reasons are the already existing optimal equivalences in SVRs which could not be improved further using PB approach and/or the regions that are aligned are conformationally different. Equivalences were preserved in majority of aSVRs. One quarter of these alignments correspond to conformationally different segments (according to the cutoff determined previously, see the first section in *Results and discussion*). An example where the scores for aSVRs fall below the cutoff and the sequences aligned are conformationally different is presented in one of the subsequent sections.

21.8% of the aSVRs have better PB scores but SDM value differences were slightly higher (3.7Å on average) than bSVRs. 25.5% of these SVRs correspond to conformationally dissimilar regions based on the cutoff previously determined; hence such regions cannot be superimposed well. In general, changes in PB-PB equivalences were observed due to re-distribution of gaps which improved the scores; however this increase is not reflected in SDM values. The short stretches of local similarity in segments of overall different conformations led to an increase in the PB scores but with a slight increase in SDM values due to poor similarity in the remaining segment presenting complex cases of superposition. This has been explained though an example illustrated in Figure 4G. The PBs *k, l and m* are aligned in aSVR, hence improving the score though the remaining segment shares low similarity. A similar observation can be made from the example in Figure 4H. The PBs *a*, *c*, *f* and *k* align in the aSVR and improve the score. Therefore where the conformations of the segments superposed are very different with similar region being very short, overall PB score may improve but the SDM values may increase slightly.

In contrast to the above scenario, 5.6% of the aSVRs have a lower PB scores but improved SDM values. 42.02% of these segments aligned are conformationally distinct. In the remaining cases, a redistribution of gaps led to different equivalences. Here, the mean difference in SDM is −16.77 for conformationally similar segments (SAP >−0.42). Small regions of similarity are preserved while the rest of alignment undergoes a change in equivalences. In certain cases, this results in an improvement of overall superposition but a decrease in PB scores. An example is presented (Figure S6A). The region of alignment of identical PBs is small (PBs *k* and *l* at the C terminal end). The rearrangement of PB equivalences in the remaining region decreases the score.

For 14 cases (0.023%) of alignments, no differences in SDM values were observed but a difference in scores for aligned PBs was found. For 12 out of 14 cases the PB score improved and for 2 cases PB score did not improve. 21.43% of these segments exhibit conformational dissimilarity. The mean difference in scores for the remaining segments corresponds to 0.52. The change in scores indicates change in PB-PB equivalences. In two-thirds of the cases, number of equivalences before and after realignment remains same without a change in overall atomic superposition. In the remaining one-thirds, the number of equivalent PBs (or % gaps in the alignment) has changed without changing the SDM values.

More surprisingly, for a limited number of cases, *i*.*e*., 3.2% of SVRs, no difference in PB scores were observed, but a change in SDM was seen. It is a consequence of new equivalences without a change in the nature of PBs aligned, which improved the SDM in 1.7% of these SVRs but did not improve in the rest 1.5% of SVRs. Finally, 7.7% of the aSVRs showed a decrease both in PB scores as well as higher deviation at Cα positions. It is mainly due to repeats of PBs which leads to the possibility of alternate alignments analogous to alignment of low complexity regions in amino acid sequences. The local similarity is observed at the ends of the alignment. PBs at the end come close while eliminating the gap which results in poor SDM and poor score (see Figure S6B). Structural alphabets *m* and *f* are repeated. As a result, many alternate alignments are possible. The PBs *c*, *f* and *k* in the segment of protein cytochrome P450 (PDB code: 1io7, chain A;[58]) could align to PBs *c*, *f*, *b or d*, *f* and *k*.

The application of the approach in modeling loop regions and in analyzing structure-function relationships has been discussed in the next two sections.

### Loop modeling

One of the most challenging tasks in comparative modeling [59], [60] is to obtain an accurate model of protein loops as they often hold the functional site [61], [62]. Errors in modeling loops are high as they are structurally variable regions and may not be conserved even among the closely related proteins [63], [64]. Modeling loop regions is difficult as the conformations also depend on length of the loop and certain key residues [65]. If the sequence similarity among the homologues is low or the regions are variable in length, the problem is compounded. Additionally, the number of geometrically possible loop conformations increases exponentially with loop length. Consequently, it becomes a daunting task to obtain an accurate model of loop regions. The conformation of a loop can be predicted by identifying a loop template from homologous structure or by searches in databases of loop conformations of various lengths obtained from known three-dimensional structures [59], [66]–[68]. It has been shown previously that the modeling of loops is more accurate if a homologue is used as one of the templates [69]. However, finding a homologue as a template for loop modeling is not always possible and in most cases a template is obtained from database search. The alternate approach, *ab initio* modeling of loop region is based on the potential or scoring function and works best for short segments [59], [70]–[72].

**Figure 5:**
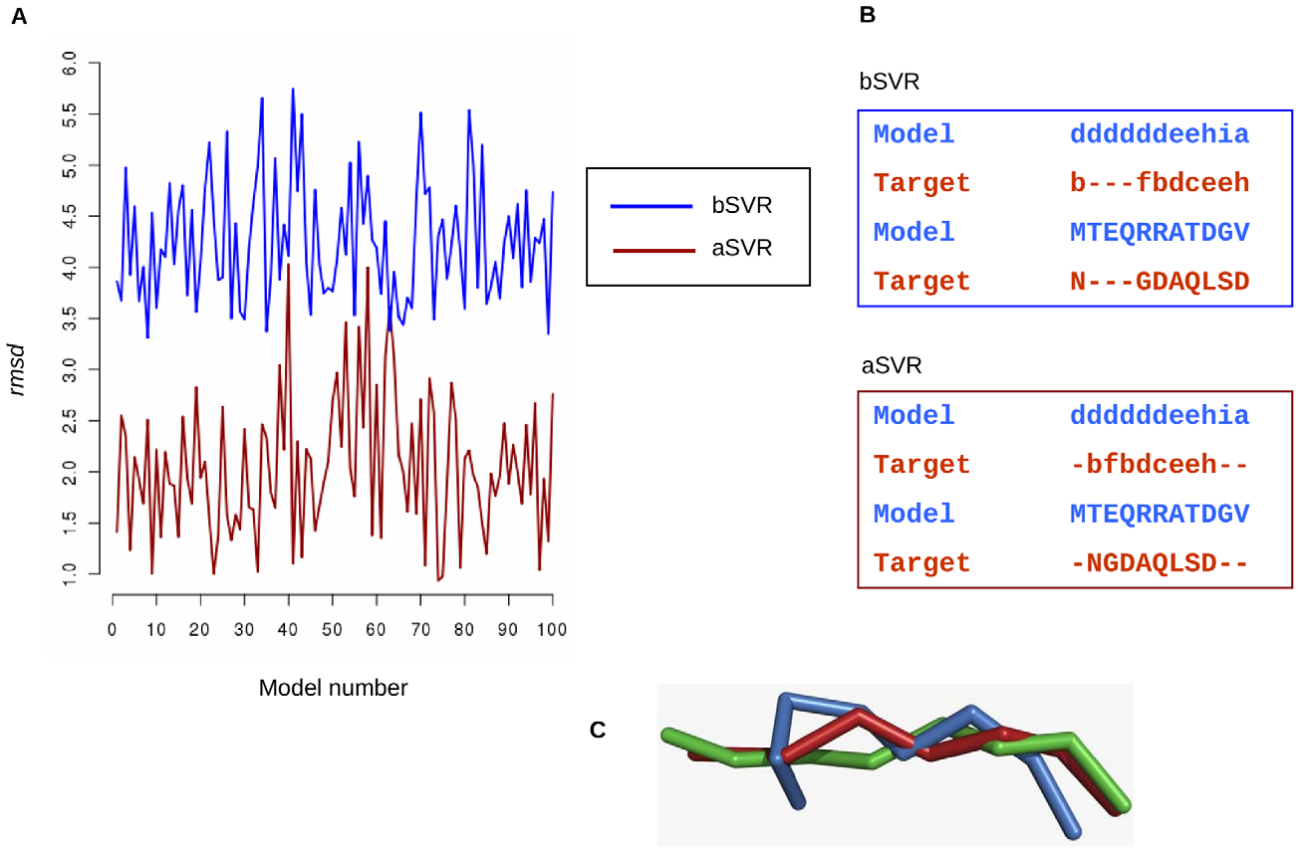
Illustrative example to highlight the utility of Protein Blocks in comparative modeling. A. Plot shows the variation in RMSD values of the models with respect to the template structure generated based on the new equivalences (aSVRs, dark red) and previousequivalences (bSVRs, blue). B. The alignments used in modeling the template fragment. The top panel shows the alignment of model and template structures (PB alignment and the corresponding amino acid sequences) based on previous equivalences. The bottom panel shows the new equivalences as obtained using our approach. C. The superposition of crystal structure for the target (green), modeled structure based on previous equivalences (blue) and new equivalences (red).

Having known that loop modeling is non-trivial and is most accurate when the equivalent regions are obtained from homologues, we have explored the use of information on local conformation through representation of templates as Protein Blocks in obtaining clues on comparative modeling. This has been exemplified through modeling exercise of a segment of Alpha-l-arabinofuranosidase protein (from Bacillus *stearothermophilus*, PDB code 1qw9 [73]) using Beta-D-xylosidase structure (PDB code 1w91 [74]) as the template which share overall sequence identity of 7.7% with the target. A number of models (100 each) were generated using Modeller 9v7 with classical approach [60] based on alignments from bSVRs and aSVRs of the target and template sequences. Figure 5A shows the variation in *rmsd* values for various models with respect to the template structure. *Rmsd* values are lower when models are generated based on the equivalences from aSVRs (red, Figure 5A) as compared to bSVRs (blue, Figure 5A). This indicates an improvement in models of the target segments when new equivalences based on PB approach were used. The two alignments: bSVR and aSVRs along with the corresponding PBs are shown in Figure 5B. For further analysis, the best models having lowest *rmsd* with respect to the template structure from each set of 100 models were selected (model 8: lower plot for aSVRs; and model 74: upper plot for SVRs). Figure 5C shows the superposition of the modeled segments with the known crystal structure (green) based on the alignment from bSVRs (blue) and aSVRs (red), respectively. The model generated based on the equivalences from PB approach produces lower *rmsd* (1.98 Å) when superposed on the crystal structure as compared to the model generated using the original approach (*rmsd*: 3.50 Å).

### Understanding sequence-structure relationships

**Figure 6:**
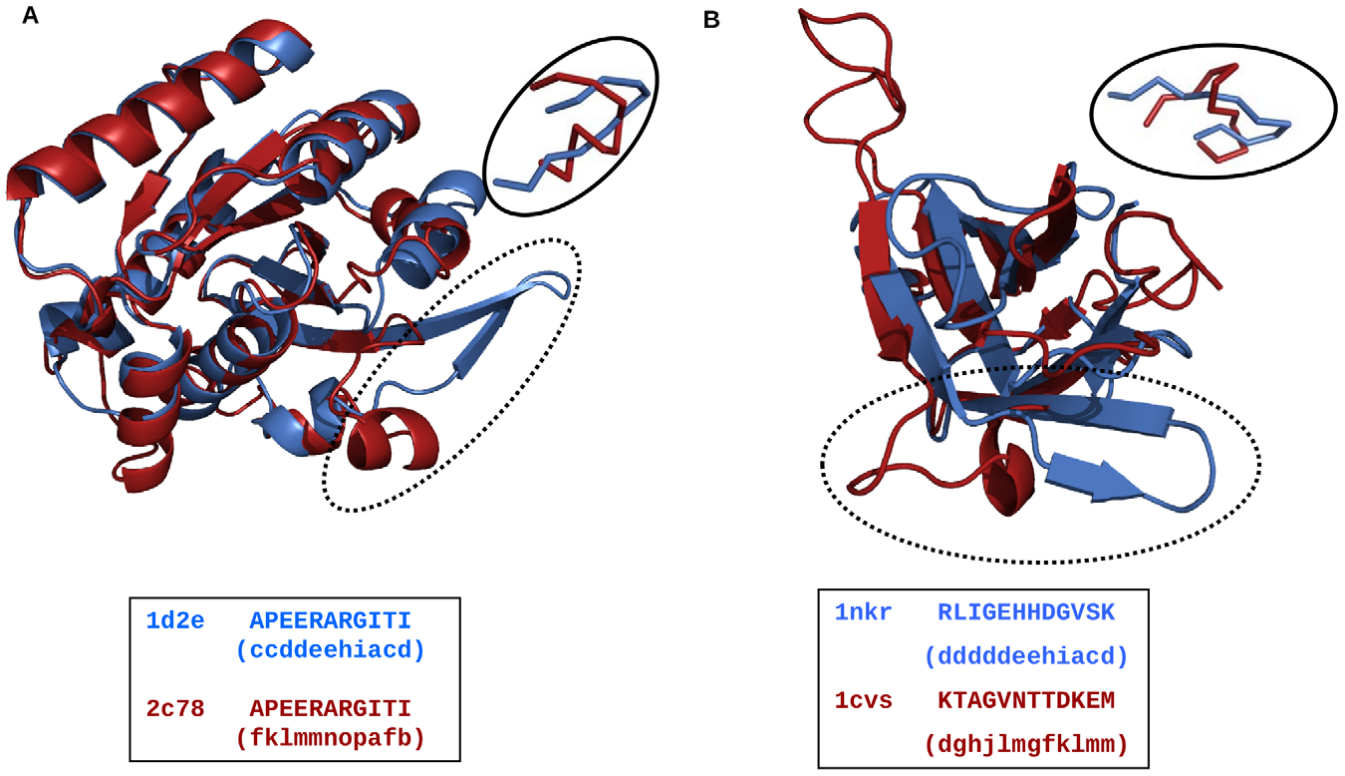
Superposition of homologous pairs of protein structures. The equivalent regions that have adopted a different structure are encircled. The inset shows the local alignment of the conformationally distinct regions. The amino acid residues and the corresponding PBs (in brackets) are shown in a box alongside. A. An illustrative example where the encircled region exhibit high sequence similarity but low structural similarity. B. A classical scenario, where the encircled region has poor sequence similarity and poor structural similarity.

Sequences of homologous proteins may evolve and diverge beyond recognition by simple homology searches. Usually, the extent of difference exhibited by sequences is higher compared to structures. In this section we show how the current analysis of consideration of PB-based alignment of SVRs can be taken to the next level of understanding of sequence-structure relationships. Here we present two examples where the local structures are very different in the pairs of homologous protein structures. Figure 6A shows the superposition of 3-D structures of two homologous elongation factors 1d2e [75] (from cow) and 2c78 [76] (from *Thermus thermophilus*) belonging to SCOP family c.37.1.8. The regions, encircled in Figure 6A are identical in terms of amino acid sequence but adopt very different structures. The PB score after optimal PB-based alignment of SVRs (aSVR) is −2.07. Figure 6B shows an example of homologous protein structures (PDB code 1nkr [77] and 1cvs [78]; SCOP family b.1.1.4, I-set domains) with poor sequence and structural similarity in a local region. Although the rest of the structures superimpose well, regions encircled in Figure 6B have very different local structures. The PB-score after the optimal PB-based alignment of SVRs is -2.07. As illustrated in two examples above, the regions are conformationally different.

In the example of elongation factors shown in Figure 6A one might expect almost identical structure for the local regions with identical sequences of two closely related proteins However the PB-based alignment of SVRs shows that this is not a spatial difference of conformationally similar SVRs. Indeed the low PB-score indicates very different conformations of identical amino acid sequence regions. In fact the extent of conformational difference between SVRs of homologues is comparable to that shown for another pair of homologous proteins in Figure 6B where the amino acid sequences in SVRs is very different [79]. Thus PB-based alignment of local regions (SVRs) are very helpful in cautioning us on unexpected structural differences even among “equivalent” SVRs of homologous proteins with highly similar or even identical amino acid sequences. Further, the example of elongation factor suggests that prediction of secondary structures based on sequence composition and sequence similarity to a ‘homologue’ should be exercised with caution. Such conformational differences are often possible in the functional regions of homologous proteins when the homologues are crystallized in different functional forms such as active and inactive forms of enzymes.

### Conclusions

In the current work, we have presented a refined view of the regions of homologous protein structures that exhibit apparent high deviation on global structural superposition. When the deviation is high, the equivalences assigned through atomic superimposition are inaccurate. Through representation of protein structures as PB sequence, conformational similarity could be identified for 159,780 (74%) variable segments, based on PB scores, an increase by 21%, compared to a classical structural alignment approach in the database of structurally aligned homologous protein structures. The improvement was also reflected in the lower SDM in 3D superposition based on new equivalences after re-alignment of SVRs. The equivalences could be refined for the capping regions of helices and strands and loops. Regions of high similarity could be located in homologous pairs of protein structures even when the aligned regions were of different lengths. Also segments which were spatially displaced could be identified and aligned efficiently. All these cases have been explained through appropriate examples. For the cases where the approach does not perform as well, the best (most optimal) alignment can be chosen based on the global context in the protein structures following the principles governing protein structure; for example, regions flanking the variable segment could be considered. The best alignment could be the one with continuous helix or strand uninterrupted by gaps in the alignment. The approach can be used in identifying equivalent regions in homologous structures that do not share structural similarity and in the understanding of sequence-structure relationship. It can aid in providing clues to model loops for which homologue of similar length is unavailable. The approach can be extended to understand the effect of amino acid substitutions on the local structural alterations in the homologous protein structures. As the approach is quite general, it can be used in conjunction with any structural alignment algorithm.

With an improvement in structural alignments which are central in understanding of protein structure-function and evolutionary relationships, the applications of the approach are manifold. The approach can be extended to refine regions of high deviation obtained using simultaneous superposition of multiple protein structures. The method can be improved by using gap penalties specific to PB types with respect to major secondary structures. In the near future, we propose to develop a web server based on our refinement approach. This comprehensive data set on homologous structures would serve as a valuable resource to study the extent and nature of alterations/structural rearrangements in backbone conformation of homologous structures as a consequence of substitutions (conservative as well as non conservative) and indels during the course of evolution.

## Materials and Methods

### Protein Data set

The protein data set was obtained from PALI [36] (Phylogeny and Alignment of homologous protein structures) v2.7 database which contains structure-based sequence alignments for protein domain families defined by SCOP database (v 1.73). The data set of 74,705 pairwise alignments, generated through DALI [14] software followed by rigid body superimposition, correspond to 1,664 protein domain families. The structural alignments were analyzed to identify topologically equivalent and non equivalent residues. A stretch of three or more contiguous residues with Cα–Cα deviation at every position lower than 3.0 Å is considered as topologically equivalent segment or Structurally Conserved Region (SCR). The other regions are considered Structurally Variable Regions (SVRs). This rule, classically used in PALI, categorizes regions as Structurally Conserved Regions or Structurally Variable Regions. Based on this criterion, 542,610 SCRs and 347,062 SVRs were identified. These SVRs correspond to 49% of the alignment positions in the data set. Of these, 215,920 complete SVRs with more than three aligned PBs have been considered for further analyses. Our entire analysis is confined to alignment of SVRs.

### Protein Blocks

Protein Blocks (PBs) correspond to a set of 16 local prototypes, labeled from *a* to *p* (see Figure 1 of ref [43]), of 5 residues length based on (φ, ψ)dihedral angles description. They were obtained by an unsupervised classifier similar to Kohonen maps [80] and hidden Markov models [81]. The PBs *m* and *d* can be roughly described as prototypes for central α-helix and central β-strand, respectively. PBs *a* through *c* primarily represent β-strand N-caps and PBs *e* and *f*, C-caps; PBs *g* through *j* are specific to coils, PBs *k* and *l* to α -helix N-caps, and PBs *n* through *p* to C-caps. This structural alphabet allows a reasonable approximation of local protein 3D structures with a root mean square deviation (*rmsd*) now evaluated at 0.42 Å [42], [43]. PBs have been assigned using in-house software. It follows rules similar to assignment done by PBE web server (http://bioinformatics.univ-reunion.fr/PBE/) [41].

### Re-alignment of structurally variable region

To re-align SVRs in quest of improvement of alignments, we have adapted our previous approach [41], [45]. We had proposed a PB substitution matrix (PB SM) similar to a matrix used for sequence alignment. A novel refined version of PB SM optimized for mining databases and improving the alignment quality has been generated (Joseph *et al.*, submitted). In this work, we have used the refined PB SM coupled with classical CLUSTALW approach [52] to realign protein structures. The parameters used in CLUSTALW were tuned to make it specific for PBs instead of amino acid residues. All residue-specific and position-specific gap penalties were turned off. A range of gap penalty values were evaluated systematically for generating alignments. Finally, a gap opening penalty of 10 and a uniform gap extension penalty of 0.2 were chosen based on the alignment scores. It must be noted that PB substitution matrix values were scaled between 0 and 10 to make it compatible with the alignment software. Newly aligned SVRs are named aSVR while previous alignments are named simply bSVR.

### Calculation of alignment scores

To evaluate the quality of new alignments of SVRs over the previous alignments, scores were computed for both alignments. Two scores were calculated for each alignment, based on inclusion or exclusion of gaps in the alignment. Calculation of these scores would reflect the differences in two alignments of a pair of segments in terms of the substitution of PB at an alignment position as well as the lengths of the alignment. The aligned PB positions were scored based on the values from PB SM. Summation of these values was normalized by the number of PB pairs to compute the Scores for Aligned Pairs (SAP) for an alignment. To calculate scores for complete alignment (SCA), including gaps, every alignment position with a gap was given a score of -3. The scores were normalized by the length of the alignment.

### Calculation of SDM values

To assess the improvement of alignments using our approach, SDM of SVR before and after re-alignment were compared. PROFIT software [54] was used to calculate *rmsd* values. The SVRs corresponding to N and C termini were removed from the analysis. 67,234 SVRs were considered for this analysis. *Rmsds* for the remaining SVRs were converted into structural distance metric (SDM) as proposed by Blundell and coworkers [55], [56].
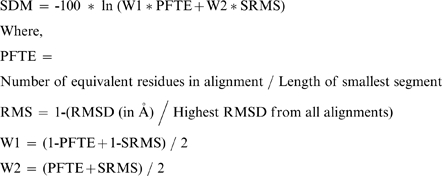
Suitable modifications have been done in SDM calculations to make it suitable for the data we present here. *i*.*e*., in RMS calculation instead of dividing *rmsd* by 3.0 we are dividing rmsd by the highest rmsd (24.97) from all the alignments for SVRs to get the values of RMS in the range of 0 to 1.

## Supporting Information

Figure S1 **The distribution of scores for bSVRs (A and C) and aSVRs (B and D).** (A) and (B) show the distribution of scores for bSVRs and aSVRs respectively, calculated by considering only the aligned PBs (SAP scores). (C) and (D) show the distribution of scores after including gaps in scoring (SCA) for bSVRs and aSVRs, respectively.
(TIF)

Figure S2 **The variation of scores for aligned PBs (SAP) and scores for complete alignment (SCA) for bSVRs (A) and aSVRs (B).**
(TIF)

Figure S3 **Difference of PBs aligned before and after re-alignment.** Positive values correspond to an improvement.
(TIF)

Figure S4 **PB correspondences.** A. Histogram of percentage of PB correspondences common in bSVRs and aSVRs. The plot depicts that about 30% of SVRs in the dataset share >95% of PB correspondences. B. Histogram of the percentage conservation of PB correspondences in bSVRs and aSVRs out of the common PB correspondences. The plot indicates that about 40% of SVRs exhibit very low and over 40% exhibit very high conservation of PB correspondences.
(TIF)

Figure S5 **Distribution of gaps.** A. The plot shows the difference in the percentage of gaps observed after re-alignment as compared to the percentage of gaps before re-alignment for a variable segment. B. The plot shows the distribution of difference in gap openings in the aSVRs as compared to the bSVRs.
(TIF)

Figure S6 **Illustrative examples of superposition of SVRs before and after alignment using PBs.** A: Reduced PB score and improved SDM B: Reduced PB scores and increased SDM.
(TIF)

Text S1 **Comparison of the distribution of SAP and SCA scores in bSVRs and aSVRs.**
(DOC)

Text S2 **Correlation of SAP and SCA scores in bSVRs and aSVRs.**
(DOC)

## References

[1] Hegyi H, Gerstein M (1999). The relationship between protein structure and function: a comprehensive survey with application to the yeast genome.. J Mol Biol.

[2] Kinch LN, Grishin NV (2002). Evolution of protein structures and functions.. Curr Opin Struct Biol.

[3] Orengo CA, Sillitoe I, Reeves G, Pearl FM (2001). Review: what can structural classifications reveal about protein evolution?. J Struct Biol.

[4] Orengo CA, Thornton JM (2005). Protein families and their evolution-a structural perspective.. Annu Rev Biochem.

[5] Thornton JM, Orengo CA, Todd AE, Pearl FM (1999). Protein folds, functions and evolution.. J Mol Biol.

[6] Lesk AM, Chothia C (1980). How different amino acid sequences determine similar protein structures: the structure and evolutionary dynamics of the globins.. J Mol Biol.

[7] Chothia C, Gerstein M (1997). Protein evolution. How far can sequences diverge?. Nature.

[8] Galperin MY, Walker DR, Koonin EV (1998). Analogous enzymes: independent inventions in enzyme evolution.. Genome Res.

[9] Andreeva A, Murzin AG (2006). Evolution of protein fold in the presence of functional constraints.. Curr Opin Struct Biol.

[10] Worth CL, Gong S, Blundell TL (2009). Structural and functional constraints in the evolution of protein families.. Nat Rev Mol Cell Biol.

[11] Murzin AG, Brenner SE, Hubbard T, Chothia C (1995). SCOP: a structural classification of proteins database for the investigation of sequences and structures.. J Mol Biol.

[12] Orengo CA, Michie AD, Jones S, Jones DT, Swindells MB (1997). CATH–a hierarchic classification of protein domain structures.. Structure.

[13] Carpentier M, Brouillet S, Pothier J (2005). YAKUSA: a fast structural database scanning method.. Proteins.

[14] Holm L, Park J (2000). DaliLite workbench for protein structure comparison.. Bioinformatics.

[15] Pisanti N, Soldano H, Carpentier M, Pothier J (2009). A relational extension of the notion of motifs: application to the common 3D substructures searching problem.. Journal of Computational Biology.

[16] Swindells MB, Orengo CA, Jones DT, Hutchinson EG, Thornton JM (1998). Contemporary approaches to protein structure classification.. Bioessays.

[17] Tyagi M, Sharma P, Swamy CS, Cadet F, Srinivasan N (2006). Protein Block Expert (PBE): a web-based protein structure analysis server using a structural alphabet.. Nucleic Acids Res.

[18] Wu C, Chen Y, Lim C (2010). A structural-alphabet-based strategy for finding structural motifs across protein families.. Nucleic Acids Res.

[19] Teilum K, Olsen JG, Kragelund BB (2009). Functional aspects of protein flexibility.. Cell Mol Life Sci.

[20] Bahar I, Lezon TR, Yang LW, Eyal E (2010). Global dynamics of proteins: bridging between structure and function.. Annu Rev Biophys.

[21] Hammes GG (2002). Multiple conformational changes in enzyme catalysis.. Biochemistry.

[22] Teague SJ (2003). Implications of protein flexibility for drug discovery.. Nat Rev Drug Discov.

[23] Godzik A (1996). The structural alignment between two proteins: is there a unique answer?. Protein Sci.

[24] Taylor WR, Orengo CA (1989). Protein structure alignment.. J Mol Biol.

[25] Lupyan D, Leo-Macias A, Ortiz AR (2005). A new progressive-iterative algorithm for multiple structure alignment.. Bioinformatics.

[26] Shindyalov IN, Bourne PE (1998). Protein structure alignment by incremental combinatorial extension (CE) of the optimal path.. Protein Eng.

[27] Sali A, Blundell TL (1990). Definition of general topological equivalence in protein structures. A procedure involving comparison of properties and relationships through simulated annealing and dynamic programming.. J Mol Biol.

[28] Ye Y, Godzik A (2003). Flexible structure alignment by chaining aligned fragment pairs allowing twists.. Bioinformatics.

[29] Menke M, Berger B, Cowen L (2008). Matt: local flexibility aids protein multiple structure alignment.. PLoS Comput Biol.

[30] Kolodny R, Koehl P, Levitt M (2005). Comprehensive evaluation of protein structure alignment methods: scoring by geometric measures.. J Mol Biol.

[31] Mizuguchi K, Deane CM, Blundell TL, Johnson MS, Overington JP (1998). JOY: protein sequence-structure representation and analysis.. Bioinformatics.

[32] Novotny M, Madsen D, Kleywegt GJ (2004). Evaluation of protein fold comparison servers.. Proteins.

[33] Russell RB, Barton GJ (1992). Multiple protein sequence alignment from tertiary structure comparison: assignment of global and residue confidence levels.. Proteins.

[34] Shatsky M, Nussinov R, Wolfson HJ (2004). FlexProt: alignment of flexible protein structures without a predefinition of hinge regions.. J Comput Biol.

[35] Hasegawa H, Holm L (2009). Advances and pitfalls of protein structural alignment.. Curr Opin Struct Biol.

[36] Balaji S, Srinivasan N (2001). Use of a database of structural alignments and phylogenetic trees in investigating the relationship between sequence and structural variability among homologous proteins.. Protein Eng.

[37] Pascarella S, Argos P (1992). Analysis of insertions/deletions in protein structures.. J Mol Biol.

[38] Reeves GA, Dallman TJ, Redfern OC, Akpor A, Orengo CA (2006). Structural diversity of domain superfamilies in the CATH database.. J Mol Biol.

[39] Williams SG, Lovell SC (2009). The effect of sequence evolution on protein structural divergence.. Mol Biol Evol.

[40] Sandhya S, Rani SS, Pankaj B, Govind MK, Offmann B (2009). Length variations amongst protein domain superfamilies and consequences on structure and function.. PLoS One.

[41] Joseph AP, Agarwal G, Mahajan S, Gelly JC, Swapna LS (2010). A short survey on Protein Blocks.. Biophysical Reviews.

[42] de Brevern AG (2005). New assessment of a structural alphabet.. In Silico Biol.

[43] de Brevern AG, Etchebest C, Hazout S (2000). Bayesian probabilistic approach for predicting backbone structures in terms of protein blocks.. Proteins.

[44] Offmann B, Tyagi M, de Brevern AG (2007). Local Protein Structures.. Current Bioinformatics.

[45] Tyagi M, Gowri VS, Srinivasan N, de Brevern AG, Offmann B (2006). A substitution matrix for structural alphabet based on structural alignment of homologous proteins and its applications.. Proteins.

[46] Tyagi M, de Brevern AG, Srinivasan N, Offmann B (2008). Protein structure mining using a structural alphabet.. Proteins.

[47] Fourrier L, Benros C, de Brevern AG (2004). Use of a structural alphabet for analysis of short loops connecting repetitive structures.. BMC Bioinformatics.

[48] Thomas A, Deshayes S, Decaffmeyer M, Van Eyck MH, Charloteaux B (2006). Prediction of peptide structure: how far are we?. Proteins.

[49] De Brevern AG, Etchebest C, Benros C, Hazout S (2007). “Pinning strategy”: a novel approach for predicting the backbone structure in terms of protein blocks from sequence.. J Biosci.

[50] Faure G, Bornot A, de Brevern AG (2009). Analysis of protein contacts into Protein Units.. Biochimie.

[51] Dudev M, Lim C (2007). Discovering structural motifs using a structural alphabet: application to magnesium-binding sites.. BMC Bioinformatics.

[52] Thompson JD, Higgins DG, Gibson TJ (1994). CLUSTAL W: improving the sensitivity of progressive multiple sequence alignment through sequence weighting, position-specific gap penalties and weight matrix choice.. Nucleic Acids Res.

[53] Zhu ZY, Sali A, Blundell TL (1992). A variable gap penalty function and feature weights for protein 3-D structure comparisons.. Protein Eng.

[54] Profit.

[55] Johnson MS, Sali A, Blundell TL (1990). Phylogenetic relationships from three-dimensional protein structures.. Methods Enzymol.

[56] Johnson MS, Sutcliffe MJ, Blundell TL (1990). Molecular anatomy: phyletic relationships derived from three-dimensional structures of proteins.. J Mol Evol.

[57] Brady RL, Dodson EJ, Dodson GG, Lange G, Davis SJ (1993). Crystal structure of domains 3 and 4 of rat CD4: relation to the NH2-terminal domains.. Science.

[58] Park SY, Yamane K, Adachi S, Shiro Y, Weiss KE (2002). Thermophilic cytochrome P450 (CYP119) from Sulfolobus solfataricus: high resolution structure and functional properties.. J Inorg Biochem.

[59] Marti-Renom MA, Stuart AC, Fiser A, Sanchez R, Melo F (2000). Comparative protein structure modeling of genes and genomes.. Annu Rev Biophys Biomol Struct.

[60] Sali A, Blundell TL (1993). Comparative protein modelling by satisfaction of spatial restraints.. J Mol Biol.

[61] Fetrow JS, Godzik A, Skolnick J (1998). Functional analysis of the Escherichia coli genome using the sequence-to-structure-to-function paradigm: identification of proteins exhibiting the glutaredoxin/thioredoxin disulfide oxidoreductase activity.. J Mol Biol.

[62] Jones S, Thornton JM (1997). Prediction of protein-protein interaction sites using patch analysis.. J Mol Biol.

[63] Fiser A, Do RK, Sali A (2000). Modeling of loops in protein structures.. Protein Sci.

[64] Fiser A, Feig M, Brooks CL, Sali A (2002). Evolution and physics in comparative protein structure modeling.. Acc Chem Res.

[65] Oliva B, Bates PA, Querol E, Aviles FX, Sternberg MJ (1997). An automated classification of the structure of protein loops.. J Mol Biol.

[66] Greer J (1980). Model for haptoglobin heavy chain based upon structural homology.. Proc Natl Acad Sci U S A.

[67] Jones TA, Thirup S (1986). Using known substructures in protein model building and crystallography.. Embo J.

[68] van Vlijmen HW, Karplus M (1997). PDB-based protein loop prediction: parameters for selection and methods for optimization.. J Mol Biol.

[69] Srinivasan N, Blundell TL (1993). An evaluation of the performance of an automated procedure for comparative modelling of protein tertiary structure.. Protein Eng.

[70] Moult J, James MN (1986). An algorithm for determining the conformation of polypeptide segments in proteins by systematic search.. Proteins.

[71] Bruccoleri RE, Karplus M (1987). Prediction of the folding of short polypeptide segments by uniform conformational sampling.. Biopolymers.

[72] Fine RM, Wang H, Shenkin PS, Yarmush DL, Levinthal C (1986). Predicting antibody hypervariable loop conformations. II: Minimization and molecular dynamics studies of MCPC603 from many randomly generated loop conformations.. Proteins.

[73] Hovel K, Shallom D, Niefind K, Belakhov V, Shoham G (2003). Crystal structure and snapshots along the reaction pathway of a family 51 alpha-L-arabinofuranosidase.. Embo J.

[74] Jakoncic J, Shoham G, Stojanoff V (2010). Crystal Structure of 1,4-Beta-D-Xylan Xylohydrolase from Geobacillus Stearothermophilus. (to be published)..

[75] Andersen GR, Thirup S, Spremulli LL, Nyborg J (2000). High resolution crystal structure of bovine mitochondrial EF-Tu in complex with GDP.. J Mol Biol.

[76] Parmeggiani A, Krab IM, Okamura S, Nielsen RC, Nyborg J (2006). Structural basis of the action of pulvomycin and GE2270 A on elongation factor Tu.. Biochemistry.

[77] Fan QR, Mosyak L, Winter CC, Wagtmann N, Long EO (1997). Structure of the inhibitory receptor for human natural killer cells resembles haematopoietic receptors.. Nature.

[78] Plotnikov AN, Hubbard SR, Schlessinger J, Mohammadi M (2000). Crystal structures of two FGF-FGFR complexes reveal the determinants of ligand-receptor specificity.. Cell.

[79] Ghozlane A, Joseph AP, Bornot A, de Brevern AG (2009). Analysis of protein chameleon sequence characteristics.. Bioinformation.

[80] Kohonen T (1982). Self-organized formation of topologically correct feature maps.. Biological Cybernetics.

[81] Rabiner L (1989). A tutorial on hidden Markov models and selected application in speech recognition.. Proceedings of the IEEE.

[82] De Lano WL (2002). The PyMOL Molecular Graphics System..

