# Supplementary figures and images for: Identification of Local Conformational Similarity in Structurally Variable Regions of Homologous Proteins Using Protein Blocks
Source: PLoS One. 2011 Mar 18;6(3):e17826. doi: 10.1371/journal.pone.0017826 (PMC3060819; doi:10.1371/journal.pone.0017826)

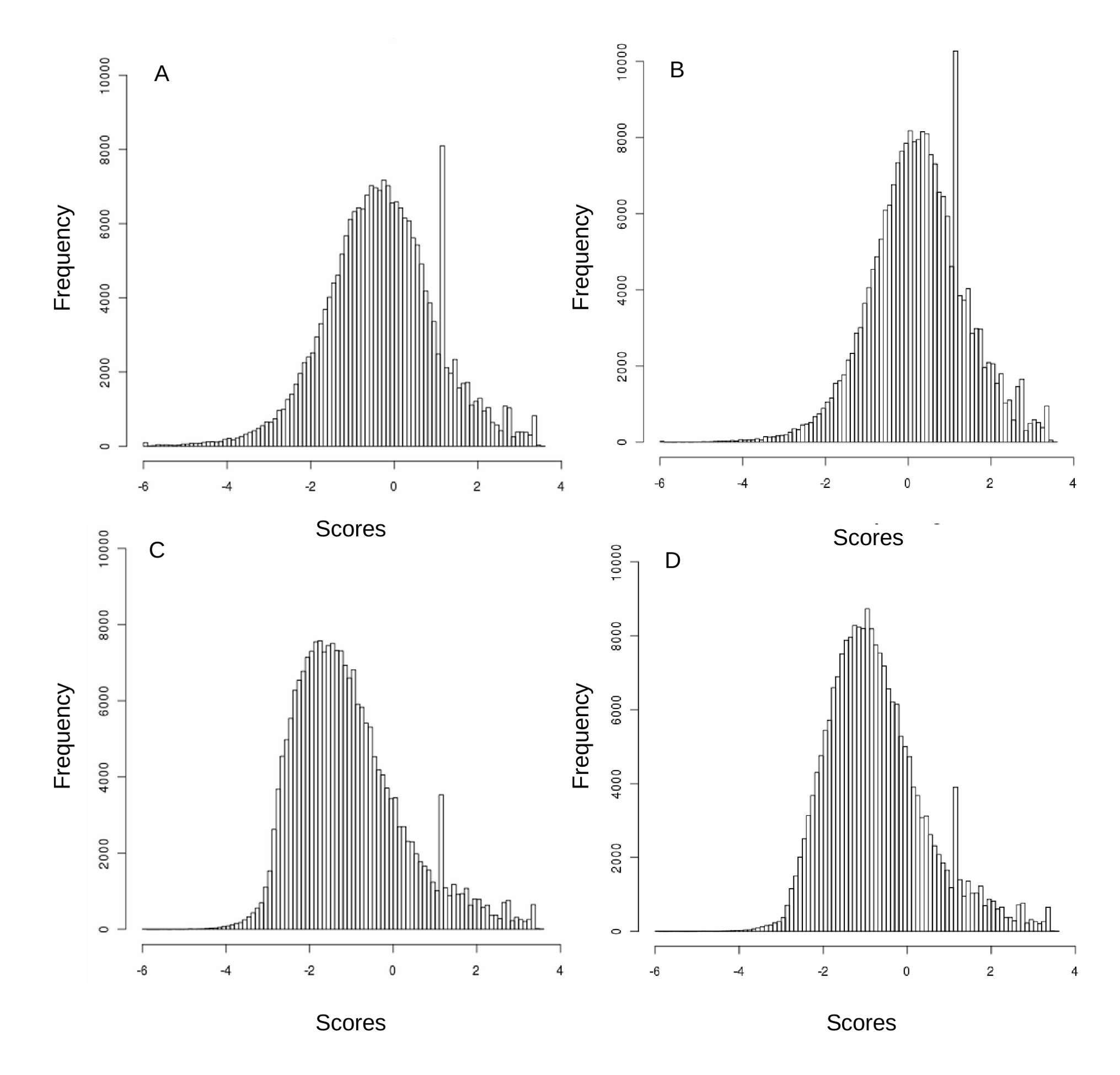

Supplement: Figure S1 — The distribution of scores for bSVRs (A and C) and aSVRs (B and D). (A) and (B) show the distribution of scores for bSVRs and aSVRs respectively, calculated by considering only the aligned PBs (SAP scores). (C) and (D) show the distribution of scores after including gaps in scoring (SCA) for bSVRs and aSVRs, respectively. (TIF) [file pone.0017826.s001.tif]

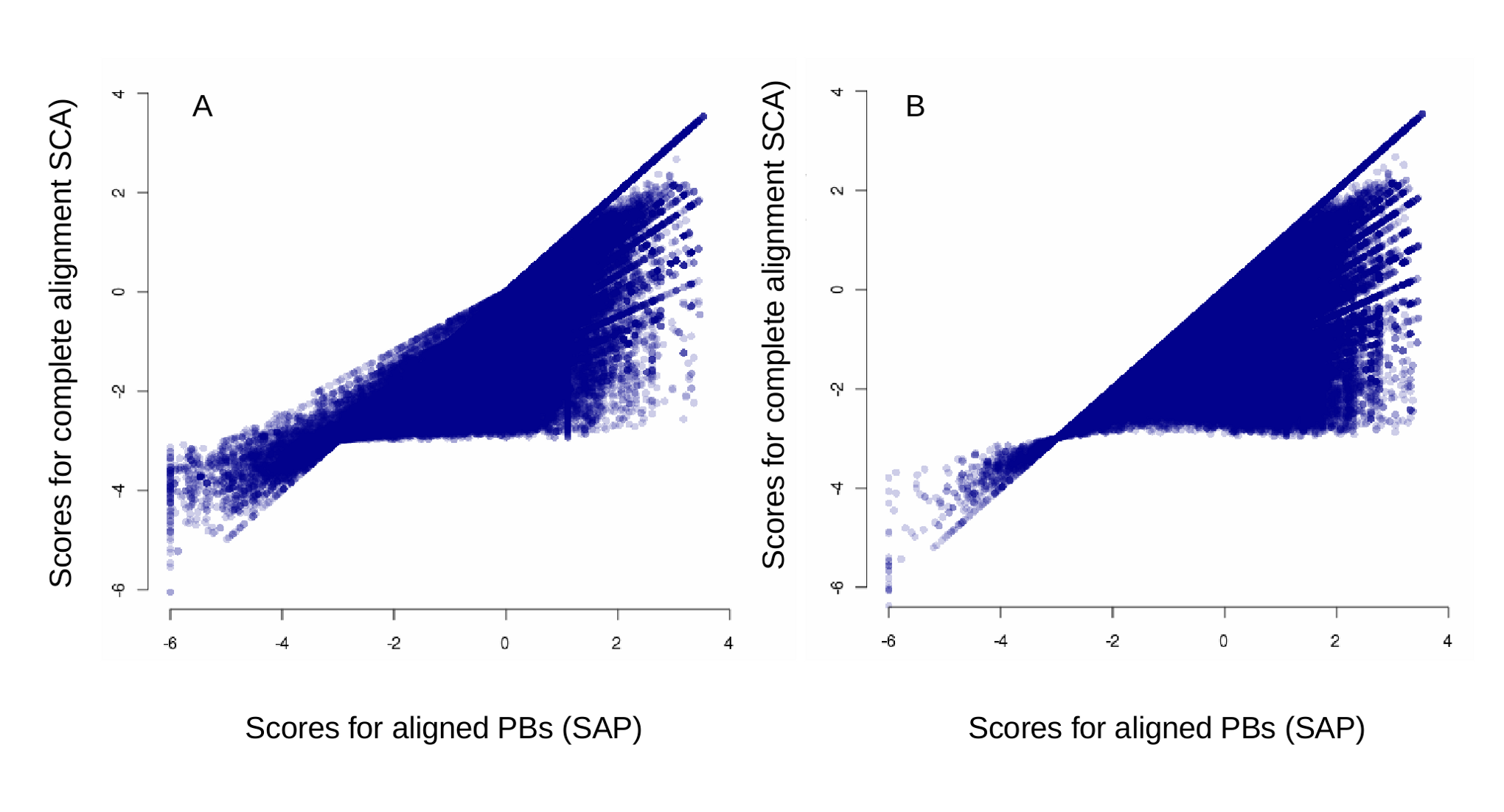

Supplement: Figure S2 — The variation of scores for aligned PBs (SAP) and scores for complete alignment (SCA) for bSVRs (A) and aSVRs (B). (TIF) [file pone.0017826.s002.tif]

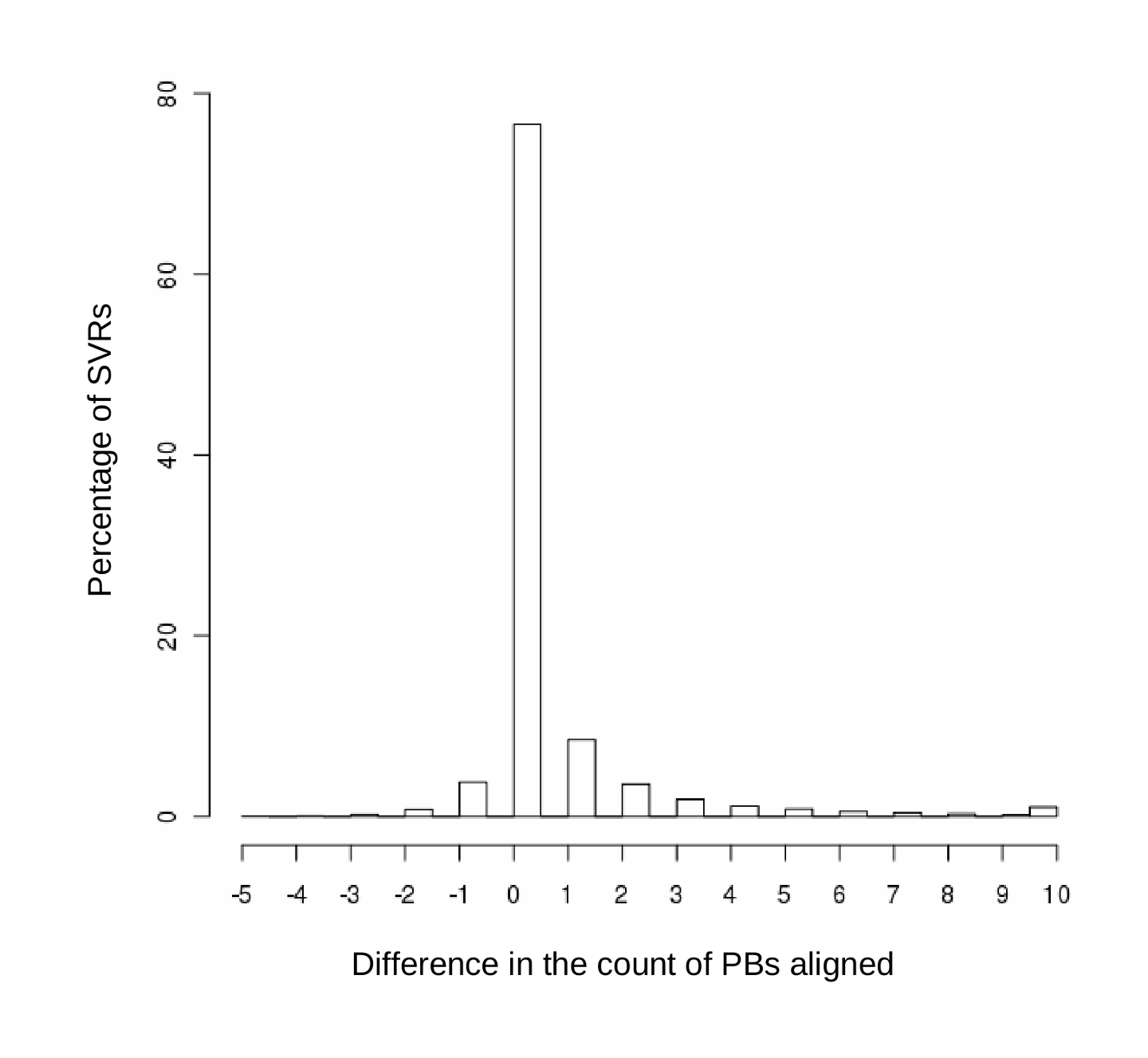

Supplement: Figure S3 — Difference of PBs aligned before and after re-alignment. Positive values correspond to an improvement. (TIF) [file pone.0017826.s003.tif]

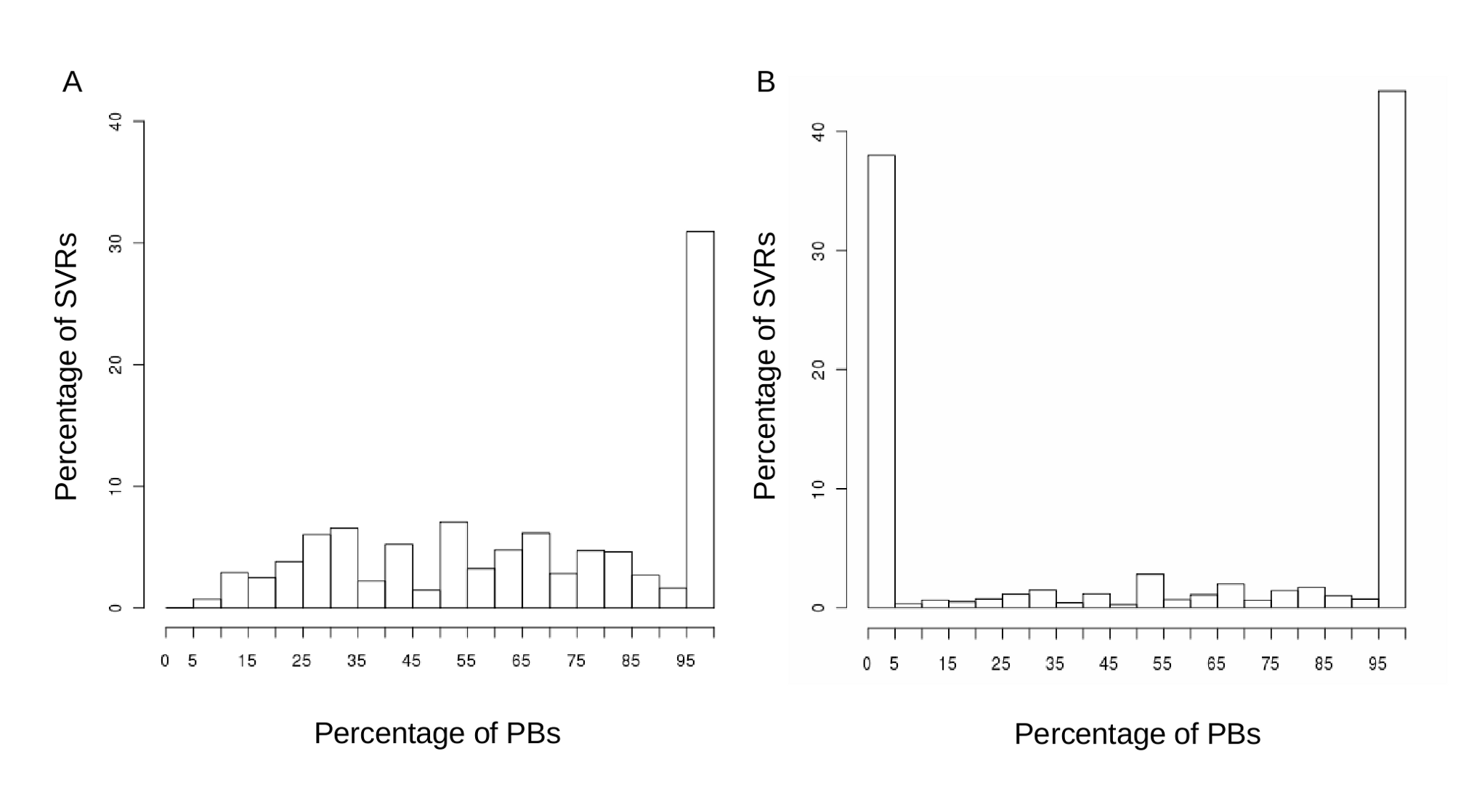

Supplement: Figure S4 — PB correspondences. A. Histogram of percentage of PB correspondences common in bSVRs and aSVRs. The plot depicts that about 30% of SVRs in the dataset share >95% of PB correspondences. B. Histogram of the percentage conservation of PB correspondences in bSVRs and aSVRs out of the common PB correspondences. The plot indicates that about 40% of SVRs exhibit very low and over 40% exhibit very high conservation of PB correspondences. (TIF) [file pone.0017826.s004.tif]

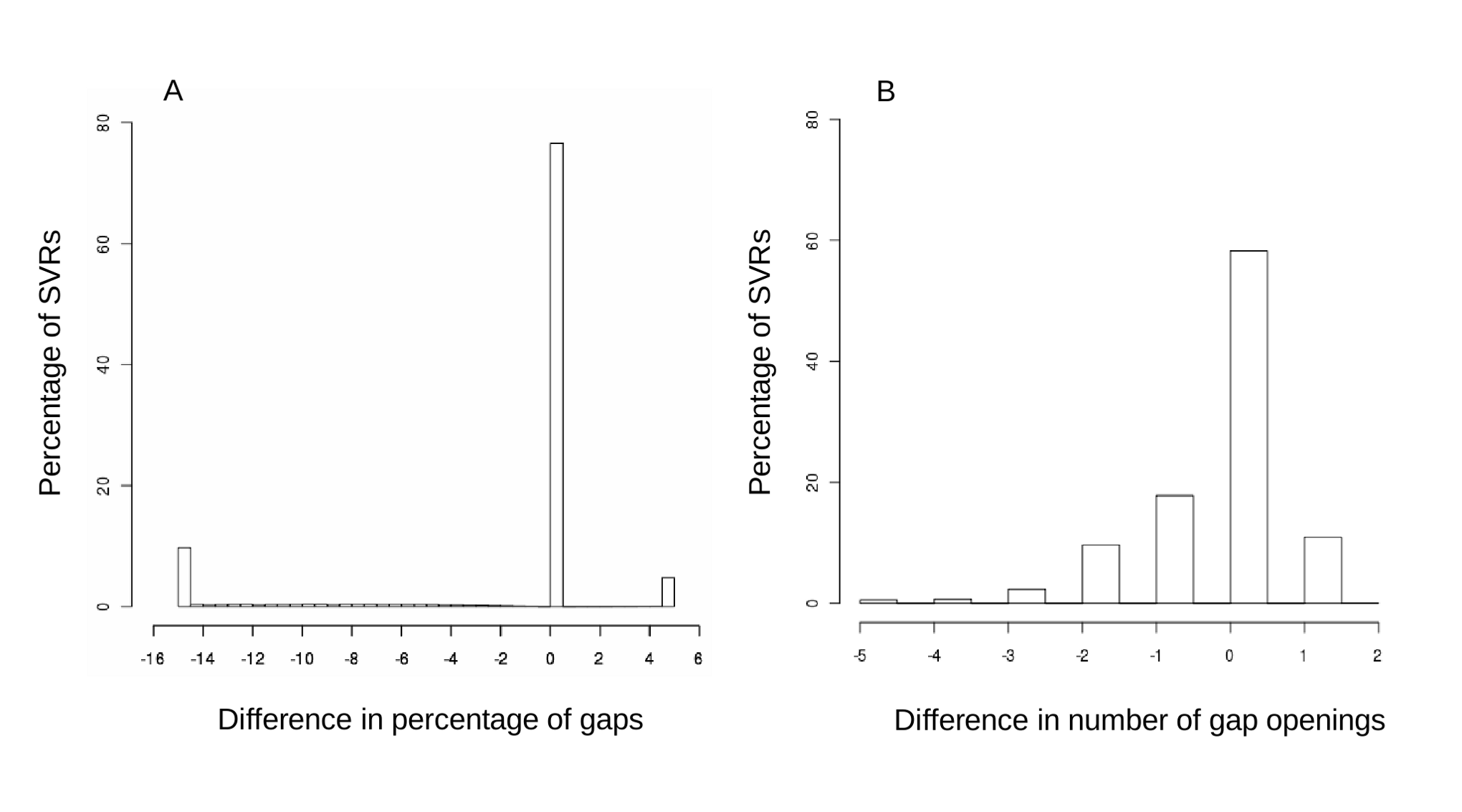

Supplement: Figure S5 — Distribution of gaps. A. The plot shows the difference in the percentage of gaps observed after re-alignment as compared to the percentage of gaps before re-alignment for a variable segment. B. The plot shows the distribution of difference in gap openings in the aSVRs as compared to the bSVRs. (TIF) [file pone.0017826.s005.tif]

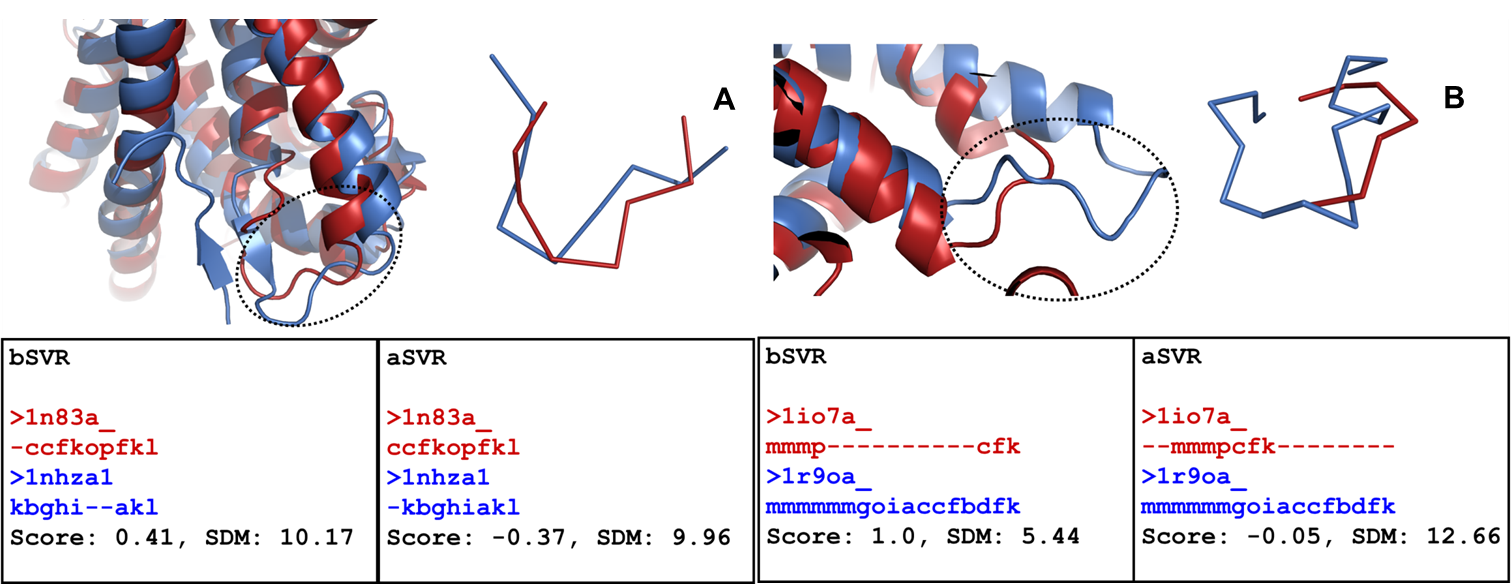

Supplement: Figure S6 — Illustrative examples of superposition of SVRs before and after alignment using PBs. A: Reduced PB score and improved SDM B: Reduced PB scores and increased SDM. (TIF) [file pone.0017826.s006.tif]
