# Supplementary material for: Identification of Local Conformational Similarity in Structurally Variable Regions of Homologous Proteins Using Protein Blocks
Source: PLoS One. 2011 Mar 18;6(3):e17826. doi: 10.1371/journal.pone.0017826 (PMC3060819; doi:10.1371/journal.pone.0017826)
Supplement: Text S1 — Comparison of the distribution of SAP and SCA scores in bSVRs and aSVRs. (DOC) [file pone.0017826.s007.doc]

**Comparison of the distribution of SAP and SCA scores in bSVRs and aSVRs**

The values for SCA and SAP were found to be normally distributed. A specific peak is observed for all scores (before and after realignment) at 1.1, See supplementary Figure S1). The bSVRs and aSVRs in this range are dominated by PB notations for β-strand and α-helix. As per our scoring scheme, an alignment of *m* and *d* with itself is scored 1.1 and 1.4, respectively which explains why a peak is observed for values between 1.1 and 1.2. The mean SAP values are respectively 0.73, -0.30 and 0.25 for SCRs (data not shown), bSVRs and aSVRs. The mean SCA values for bSVRs and aSVRs are significantly different going originally from -1.06 to -0.73 respectively (Figure S1).
