# Supplementary material for: Identification of Local Conformational Similarity in Structurally Variable Regions of Homologous Proteins Using Protein Blocks
Source: PLoS One. 2011 Mar 18;6(3):e17826. doi: 10.1371/journal.pone.0017826 (PMC3060819; doi:10.1371/journal.pone.0017826)
Supplement: Text S2 — Correlation of SAP and SCA scores in bSVRs and aSVRs. (DOC) [file pone.0017826.s008.doc]

**Correlation of SAP and SCA scores in bSVRs and aSVRs**

As expected correlation between SAP and SCA scores is good for bSVRs (0.85) and aSVRs (0.70), An increase by 14% in the data set points towards higher scores (above -1) was observed (see Supplementary Figure S2). The dataset points on the diagonal correspond to alignments without gaps and therefore with identical scores. Deviation from diagonal is indicative of alignments having sequences with variable lengths. The extent of drift from the diagonal is dependent on the nature of PBs aligned as reflected from their substitution scores as well as the differences in the lengths of sequences in the alignment. A high score for aligned PBs but poor total score indicates a high similarity in the PB substitutions but with large number of gaps in the rest of the alignment; a possibility of insertion/deletion events in the equivalent region while retaining local conformational similarity. On the contrary, low score for aligned PBs would suggest an alignment of very different backbone structures which is indicative of large conformational difference in the equivalent regions of homologous proteins. The plots indicate an improvement in scores, an increase by 14% for segments scoring above -1 and a decrease by 16% in the segments scoring below -1. We observe a higher score for aSVRs compared to bSVRs indicating a higher local similarity after re-alignment and an improvement in the alignment.
